# Whole-Exome Sequencing Implicates *SCN2A* in Episodic Ataxia, but Multiple Ion Channel Variants May Contribute to Phenotypic Complexity

**DOI:** 10.3390/ijms19103113

**Published:** 2018-10-11

**Authors:** Neven Maksemous, Robert A. Smith, Heidi G. Sutherland, Hugo Sampaio, Lyn R. Griffiths

**Affiliations:** 1 Genomics Research Centre, Institute of Health and Biomedical Innovation (IHBI), School of Biomedical Sciences, Queensland University of Technology (QUT), Q Block, 60 Musk Ave, Kelvin Grove Campus, Brisbane 4059, QLD, Australia; n.maksemous@qut.edu.au (N.M.); r157.smith@qut.edu.au (R.A.S.); heidi.sutherland@qut.edu.au (H.G.S.); 2 Department of Women and Children’s Health, Randwick Campus, University of New South Wales, Randwick 2031, NSW, Australia; hugo.sampaio@health.nsw.gov.au; 3 Sydney Children’s Hospital, Randwick 2031, NSW, Australia

**Keywords:** episodic ataxia, *SCN2A*, *KCNC2*, *SCN8A*, whole-exome sequencing, acetazolamide

## Abstract

Although the clinical use of targeted gene sequencing-based diagnostics is valuable, whole-exome sequencing has also emerged as a successful diagnostic tool in molecular genetics laboratories worldwide. Molecular genetic tests for episodic ataxia type 2 (EA2) usually target only the specific calcium channel gene (*CACNA1A*) that is known to cause EA2. In cases where no mutations are identified in the *CACNA1A* gene, it is important to identify the causal gene so that more effective treatment can be prioritized for patients. Here we present a case of a proband with a complex episodic ataxias (EA)/seizure phenotype with an EA-affected father; and an unaffected mother, all negative for *CACNA1A* gene mutations. The trio was studied by whole-exome sequencing to identify candidate genes responsible for causing the complex EA/seizure phenotype. Three rare or novel variants in Sodium channel α2-subunit; *SCN2A* (c.3973G>T: p.Val1325Phe)*,* Potassium channel, Kv3.2; *KCNC2* (c.1006T>C: p.Ser336Pro) and Sodium channel Nav1.6; *SCN8A* (c.3421C>A: p.Pro1141Thr) genes were found in the proband. While the *SCN2A* variant is likely to be causal for episodic ataxia, each variant may potentially contribute to the phenotypes observed in this family. This study highlights that a major challenge of using whole-exome/genome sequencing is the identification of the unique causative mutation that is associated with complex disease.

## 1. Introduction

Hereditary episodic ataxias (EAs) are a complex group of neurological disorders usually characterized by attacks of imbalance and incoordination, often associated with progressive ataxia [1]. Weakness, dystonia, and ataxia may present between episodes. EA are clinically and genetically heterogeneous. To date, eight types of EA have been described; the dominant forms are EA1 (OMIM, 160120) and EA2 (OMIM, 108500), caused by mutations in *KCNA1* (OMIM, 176260) and *CACNA1A* (OMIM, 601011), respectively. EA1 is characterized by short episodes of ataxia (minutes) and interictal myokymia (continuous muscle movement), while EA2 is characterized by longer ataxic episodes (hours) and interictal nystagmus (rapid involuntary movements of the eyes). The onsets for both EA1 and EA2 episodes are typically in the first or second decade in life and are usually triggered by physical and emotional stress. EA2 attacks can be dramatically relieved with acetazolamide (AZ).

We have previously demonstrated the viability of a targeted Next Generation Sequencing (NGS) panel to identify known and novel causative mutations in *CACNA1A* in a group of 31 EA2 sufferers [2]. Despite the success of the method, only 48% of the screened cases could be molecularly diagnosed. Whole-Exome Sequencing (WES) has emerged as an effective diagnostic tool in identifying disease-associated genes as it captures and sequences most of the protein-coding DNA (exonic regions) of the human genome. This enables easier identification of new genes underlying neurological disorders, as well as improving diagnosis of complex and genetically heterogeneous clinical phenotypes such as EA.

Here we described two affected family members (father and daughter) diagnosed with EA with variable response to AZ treatment. Screening the *CACNA1A* gene using our targeted gene panel did not reveal any causative mutations or variants. Therefore, we conducted WES on two affected family members as well as the unaffected mother, and we used paternal grandparents’ DNA for segregation analysis purposes. Rare or novel damaging variants in multiple sodium and potassium ion channel genes were identified in the proband.

## 2. Results

WES of the EA-affected father and daughter and unaffected mother was performed on the Ion Proton platform (Thermo Fisher, Scoresby, Australia), and the data generated by Ion Proton was analyzed using Torrent Suite v5.0.2. (Thermo Fisher, Scoresby, Australia)), WES produced an average of 93× read depth. Following the completion of the trio WES chip data analysis and variant filtering prioritization, the affected father and daughter were found to carry two novel non-synonymous amino acid changing variants in common, in two ion channel genes Sodium channel α2-subunit; *SCN2A* (c.3973G>T: p.Val1325Phe) and Potassium channel, Kv3.2; *KCNC2* (c.1006T>C: p.Ser336Pro) (Table 1), while neither were present in the unaffected mother.

**Figure 1:**
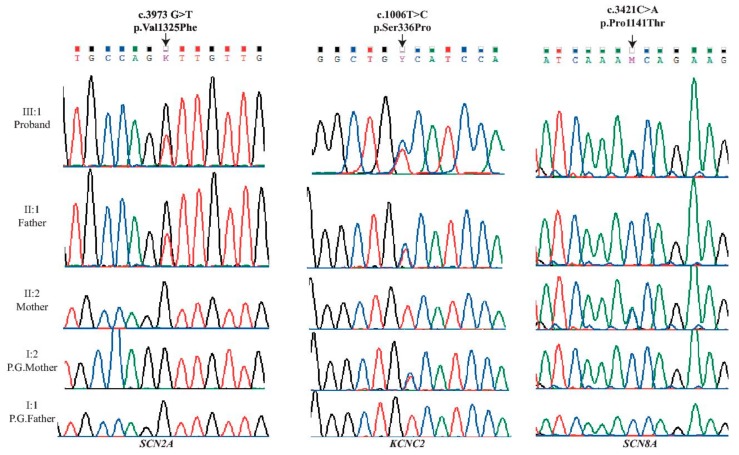
Sequencing of three novel mutations identified in Sodium channel α2-subunit (*SCN2A-left*), Potassium channel, Kv3.2 (*KCNC2-middle*) and Sodium channel Nav1.6 (*SCN8A-right*) genes. Data were obtained by Chromas (from Sanger sequencing) and the Integrative Genomics Viewer (from the WES) during the confirmation process. The three heterozygous exonic point variants; left c.3973G>T: p.Val1325Phe in exon 21 of the *SCN2A* gene, middle c.1006T>C: p.Ser336Pro in exon 3 in the *KCNC2* gene; and right c.3421C>A: p.Pro1141Thr in exon18 in the *SCN8A* gene; All were sequenced in the five family members.

Segregation analysis of the *SCN2A* and *KCNC2* variants by Sanger sequencing, showed that the c.3973G>T variant in *SCN2A* was confirmed as de novo in the proband’s father, while the c.1006T>C variant in *KCNC2* was detected in the asymptomatic paternal grandmother’s DNA. Neither of the *SCN2A* and *KCNC2* variants were detected in the proband’s mother nor paternal grandfather (Figure 1).

Moreover, due to the maternal family history of seizures, further variant filtering was conducted using Ion Reporter v4.6 (Thermo Fisher, Scoresby, Australia) to filter in variants that were potential candidates for the disease gene in the affected proband and the unaffected mother. The mother and the daughter were found to carry a very rare missense variant in Sodium voltage-gated channel alpha subunit 8 *SCN8A* (c.3421C>A: p.Pro1141Thr, 4/237332 alleles in gnomAD). Sanger sequencing also confirmed the presence of the *SCN8A* variant in both the proband and her mother only (Table 1 and Figure 1).

**Figure 2:**
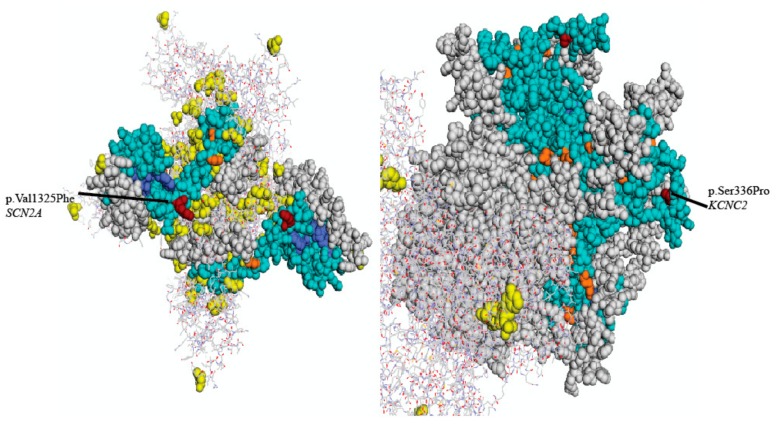
Three-dimensional structural homology model of the available structural modelling of sodium channel α2-subunit Nav1.2 (**left**) and the voltage-gated potassium channel, Kv3.2 (**right**) with mapped EA-related mutations. Arrows represent two residues *SCN2A* (p. Val1325Phe, **left**) and *KCNC2* (p.Ser336Pro, **right**) associated with EA-related symptoms.

In silico analysis using SIFT [3], PolyPhen2 [4], Mutation Taster [5], and PROVEAN [6], suggested that the three identified variants are all predicted to have a deleterious effect on protein function (Table 1). These results suggest that one or more genes might be implicated with the early onset, non-acetazolamide responsive and complicated EA symptoms of our proband. Visualizations for the SCN2A and *KCNC2* variants based on the closest available crystal structure in Mutation Assessor can be seen in Figure 2 [7]. No appropriate crystal structure was available for SCN8A.

## 3. Discussion

Targeted NGS custom panels have led to increasing the diagnostic yield in our laboratory in suspected inherited neurological diseases such as EA2 [2]. However, not all cases could be genetically diagnosed, with an overall detection rate of 48%. This may be due to the limited number of genes included on the custom-designed panel, added to the clinical overlap of EA2 with other neurogenetic syndromes, which may mislead molecular diagnosis. Therefore, WES was proposed as a solution to these problems.

In our study, we identified a novel *SCN2A* mutation in the proband and her affected father, expanding the phenotypic spectrum of *SCN2A* mutations. This *SCN2A* mutation has not been reported previously in individuals with disease in the 1000 Genomes, gnomAD and Exome Aggregation Consortium (ExAC) databases or the available literature and was confirmed as de novo in the affected father with a variable responsiveness to AZ treatment.

*SCN2A* encodes the voltage-gated sodium channel α2-subunit Na_v_1.2 which maps to chromosome 2q21-q33 in humans [8]. *SCN2A* is a member of the sodium channel family (consisting of fourteen members, including, *SCN1A*, *SCN3A* and *SCN8A*) that are widely expressed in neurons of the central nervous system (CNS) and implicated with seizure disorders. While the specific locus of the variant detected in our study has not been implicated in any symptom set, mutations in the same extracellular domain just upstream of the ion selectivity region have been implicated in seizures and autism spectrum disorder [9]. Previous studies reported different mutations in *SCN2A* that were associated with a diversity of clinical presentations i.e., benign familial infantile seizures (BFNIS), Dravet syndrome (DS), generalized epilepsy with febrile seizures and repetitive encephalopathy [10]. Previous studies also reported several *SCN2A* mutations associated with EA [11,12,13], indicating that mutations in voltage-gated sodium channels have diverse effects and can be associated with a variety of diseases. Most *SCN2A* mutation patients have seizures, and both of our two patients have recently experienced several seizure attacks. A previous study by Leach and colleagues reported a de novo *SCN2A* mutation associated with EA presentation which was responsive to AZ [11]. The authors additionally identified a potassium channel gene (*KCNQ3*) variant which was considered “neutral” according to multiple in silico algorithms and the presence of this variant in an asymptomatic family member. Similarly, in our study, a novel heterozygous missense variant was also identified in a potassium voltage-gated channel (*KCNC2*); the variant was inherited from the unaffected paternal grandmother but was predicted to be damaging at the protein level. It is possible that additional ion channel variants may moderate phenotypic range or severity.

*KCNC2* encodes the voltage-gated potassium channel, K_v_3.2, located at chromosome 12q21.1. It is also highly expressed in the brain [14]. Heterozygous mutations in the *KCNA1* gene (OMIM 176260) also located on chromosome 12 (12p13.32) have been implicated with autosomal dominant EA with myokymia (EA1) (OMIM; 160120). Additionally, a study in a family showing developmental delay and cerebellar ataxia showed a large-scale deletion resulting in the loss of exons 3-5 of *KCNC2* and the complete loss of the adjacent *ATXN7L3B* [15]. This previously reported *KCNC2* variant is a more severe alteration to gene structure than the one identified in our study, and furthermore the *KCNC2* variant we identified is also carried in the asymptomatic paternal grandmother of the proband. The variant itself is not in any specific characterized domain of the protein but is located just outside of a transmembrane region and is presumably located in an extracellular domain, which is supported by its position in the closest available structure found by Mutation Assessor (Figure 2). Unfortunately, it does not appear that this region’s function has been characterized, so there are no obvious implications for the variant’s functional effects, if any. Thus, it is unclear as to whether the variant we have identified is neutral, has incomplete penetrance, or alters the effect of the de novo *SCN2A* variant detected in the proband and the proband’s father.

EA that lacks myokymia but is associated with nystagmus is usually linked to mutations in the *CACNA1A* (OMIM; 601011) gene which cause 95% of EA2 (OMIM; 108500). AZ treatment has been effective for both EA1 [16,17] and EA2 [18]. In *SCN2A* mutation carriers, AZ has showed a variable effects; in one case AZ prevented further episodes of ataxia [11], while two studies by Liao et al. and Schwarz et al. AZ showed no effect on EA [12,13]. Thus, obtaining specific molecular diagnoses is important, as it may aid in treatment selection where the AZ or other treatment response profile of the variant is known. The proband and her affected father, who carry missense mutations in both *SCN2A* and *KCNC2*, had variable responses to AZ treatment. Although in both the proband and her father, a pediatric neurologist had noted a dramatic beneficial effect of AZ on at least one occasion, the degree of this effect was intermittent and variable, often leading to doubt about the diagnosis. Moreover, the proband showed an early onset of complex neurological signs at the age of 21 months with a poorer response to AZ treatment compared to her father, whose symptoms started at 14 years old. This suggests modifying factors may influence the severity of the proband’s symptoms and poor response to AZ treatment. Therefore, the presence of a potentially damaging *SCN8A* variant that had been inherited maternally may also play a role in the proband’s phenotype.

*SCN8A* encodes the voltage-gated sodium channel Na_v_1.6 which is mapped to chromosome 12q13 and it is highly expressed in the brain. A novel heterozygous variant in the *SCN8A* gene was found in the proband and her asymptomatic mother; nucleotide substitution c.3421C>A: p.Pro1141Thr, which alters an evolutionarily conserved amino acid. Mutations in *SCN8A* have previously been reported in association with cognitive behavioral deficits, pancerebellar atrophy ataxias and early onset of childhood epilepsies [19]. Also, mild phenotypes and incomplete penetrance have been reported for the same *SCN8A* mutations and within the same family [19,20,21]. It is interesting to note that the variant we have detected is in the first group of transmembrane repeats, which has the highest concentration of recurrent epilepsy associated mutations [22]. Despite this, its location very early in these repeats seems to have relatively fewer disease-associated variants, which may indicate a potentially weaker effect on phenotype for variants in this region that do not usually provoke medical investigation [23]. Bagnasco et al. reported Carbamazepine (CBZ), a Na^+^ channel blocker, as a successful treatment for seizures associated with very early onset of movement disorders for *SCN8A* mutation carriers [20]. We suggest that the *SCN8A* variant may contribute to the early onset and severity of the proband’s phenotypes, as well as poor response to AZ treatment. Introduction of a low dose of CBZ should be considered for the affected proband.

Since *SCN2A* and *SCN8A* mutations have been previously reported in patients with EA [11,12,13,19,20], we propose that both gene variants are likely to contribute to the complex phenotype of the proband, including lack of response to AZ. Based on the *SCN2A* de novo status, segregation analysis, and American College of Medical Genetics and Genomics guideline (ACMG) [24], we hypothesize that the *SCN2A* variant is causal for EA in the father, but that the *KCNC2* variant may also contribute to his phenotype due to overlapping physiological interactions between SCN2A (Nav1.2) and KCNC2 ion channels. In support of this hypothesis, co-expression of *SCN2A* and *KCNC2* has been identified between the two channels in the StemcellDB human pluripotent stem cell expression database using the GeneMANIA prediction server (http://genemania.org) [25], indicating that at least at some stage of development both genes are involved in overlapping physiological processes. Data from the Genotype-Tissue Expression (GTEx) project (dbGaP Accession phs000424.v7.p2) shows that *SCN2A* and *KCNC2* are co-expressed in various regions of the brain, including the cortex, frontal cortex, anterior cingulate cortex, and amygdala. However, while *KCNC2* shows little cerebellar expression *SCN2A* is highly expressed in the cerebellum, which is responsible for control of coordination and balance, providing further support for the *SCN2A* variant causing ataxia. Attack initiation may arise from a cortical spreading depression-like effect that commences in regions where *SCN2A* and *KCNC2* are co-expressed, but with possible additional effects coming from the motor cortex. It is also interesting to note that an existing animal model of sodium channel family-based periodic paralysis disorder explicitly involves interaction between sodium and potassium ions in attack triggering and a similar mechanism may be at work where both variants are present [26]. However, predicting risk or outcome of channelopathies from deleterious ion channel mutations is challenging and further investigation of the potential mechanisms of these two genes in the pathogenesis is needed [27]. This study brings into focus the roles of the three genes (*SCN2A*, *KCNC2* and *SCN8A*) in human disorders including EA and seizure. Future functional analysis of these variants in cell lines or an animal model would be of great value for understanding the pathogenicity, penetrance, and potential epistasis of these mutations.

## 4. Materials and Methods

### 4.1. Subject

**Figure 3:**
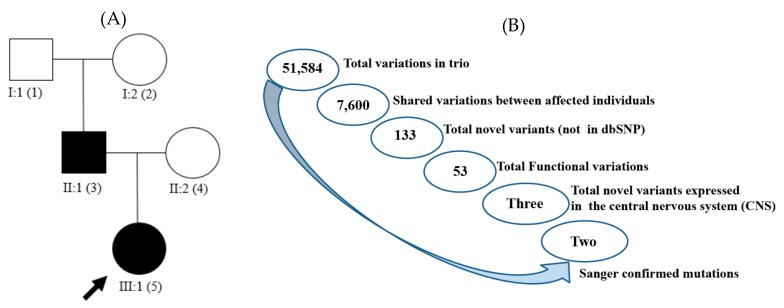
(**A**) Family tree of two affected individuals with episodic ataxia. Squares represent men; circles, women; black filled figures, affected EA; unfilled figures, unaffected; arrow represents our proband; (**B**) schematic representation of the variant filtering. Total variant counts at each stage of filtering using Ion Reporter software resulted in two non-synonymous novel variants, in the affected individuals, of genes expressed in the central nervous system and confirmed with Sanger sequencing method.

The two affected members of the family, a father and daughter (proband), underwent clinical assessments and both were diagnosed to have EA2 that lacked genetic confirmation. Both affected family members were tested using a diagnostics NGS multi-gene panel which includes complete exonic sequencing of the gene implicated in EA2, *CACNA1A*, as well as other monogenic migraine disorder genes (*ATP1A2*, *SCN1A*, *NOTCH3*, and *KCNK18*) [2]. Blood was previously collected from the affected members and referred to the Genomics Research Centre, Queensland University of Technology for molecular genetics testing. The healthy mother and paternal grandparents were recruited for segregation analysis (Figure 3A). Written informed consent for research was received from all participants and daughter’s guardians. The study was conducted in accordance with the Declaration of Helsinki, and the protocol was approved by the Human Research Ethics Committee of the Queensland University of Technology (1400000748, 20 July 2015).

### 4.2. Clinical Context

A 26-year-old man (Father) referred for testing was first noted with episodes of ataxia at age 14 years with no sign of progressive cerebellar ataxia, as he was completely asymptomatic between episodes. His clinical picture was complicated by learning problems at school, and by episodes of severe migraine, including one severe bout associated with hemiparesis following a mild head injury. A pediatric neurologist diagnosed EA2 without genetic confirmation when he was a teenager and the symptoms responded variably to AZ treatment. Genetic analysis of the family occurred when the daughter (proband) presented aged 21 months with severe and confirmed period of ataxia following a minor head injury in a fall from a couch. A cerebral CT scan without contrast was normal. There was no loss of consciousness but marked truncal ataxia and several episodes of vomiting persisted for hours after the event, and AZ treatment was commenced. There was no history of intrauterine hazards and the neonatal period had been uncomplicated. As the girl had a maternal family history of seizures (though the mother herself is not known to be affected) and reports of the proband’s subsequent symptoms varied and might have included paroxysmal involuntary movements or loss of awareness, several possible diagnoses were entertained, including EA and generalized seizures.

### 4.3. Whole-Exome Sequencing

WES was performed on genomic DNA of the EA2 diagnosed father and daughter (proband) and her unaffected mother. Libraries were prepared using the Ion AmpliSeq Exome RDY library preparation kit (Cat. no. 4489837 Rev A.0, (Thermo Fisher, Scoresby, Australia) according to the manufacturer’s protocol. The libraries were sequenced on the Ion Proton sequencer (Thermo Fisher, Scoresby, Australia). Sequence reads were aligned to the human reference genome (hg19), single-nucleotide variants and indels were called using the Ion Torrent Suite software. The bam format file generated by the Torrent Suite was uploaded and visualized for human examination using Integrative Genomics Viewer (IGV) 2.3 software (http://www.broadinstitute.org/igv) [28]. The locally hosted Ion Reporter software 4.6 (Thermo Fisher, Scoresby, Australia) was used to perform automated variant annotation and filtering. We focused on variants altering protein-coding regions and canonical splice-sites in ion channel genes and other genes plausibly linked with the phenotype. 51,584 variants were detected in a total of 14,911 genes in the trio. To select the candidate variants iterative filtering was performed based on a workflow designed for the present study (Figure 3B). Ion Reporter v4.6 was used to filter in variants which were potential candidates for the disease gene in the affected family members. All 7600 variations shared between the affected father and daughter were filtered in and variations that were found in the registered databases dbSNP (www.ncbi.nlm.nih.gov/projects/SNP/Build138), and 1000 Genomes Project (www.1000genomes.org) were excluded, which resulted in a total of 133 common to the father and daughter. Additional filtering selected the variants with functional effect (altering amino acid composition of proteins), resulting in 53 candidate variants in common between them. As a final step, all the remaining variants common to father and daughter in genes known to be expressed in the CNS were selected, which narrowed the list to three non-synonymous variants. These variants were subsequently confirmed or rejected by Sanger sequencing, leaving only two variants in *SCN2A* and *KCNC2* common to the father and proband (Figure 1 and Table 1). Following mutation confirmation, segregation analysis of identified variants was performed by Sanger sequencing in the proband, her parents, and her paternal grandparents.

## 5. Conclusions

Applying WES in two undiagnosed affected family members with EA-like phenotypes identified a variant in *SCN2A* as the likely cause, although two additional potential disease-causing variants in some family members in two different ion channel genes (*KCNC2* and *SCN8A*) may also contribute to phenotypic complexity. Identification of the *SCN2A* and *SCN8A* ion channel gene variants which have been associated with the response to AZ or CBZ treatment, respectively, should increase our understanding of the causes and the pathophysiology of this EA-seizure condition. Additionally, inclusion of these genes in testing panels or analysis parameters in cases of familial or sporadic ataxias or seizure related disorders should be considered if first line genes are not implicated by initial testing.

## Figures and Tables

**Table 1:** Overall results of whole-exome sequencing.

Locus	Gene	Exon	Protein	Coding	SIFT	Polyphen	Mutation Taster	PROVEAN
chr2:166231195	*SCN2A*	21	p.Val1325Phe	c.3973G>T	D (0.0)	D (1)	D	D (−4.5)
chr12:75444779	*KCNC2*	3	p.Ser336Pro	c.1006T>C	D (0.023)	D (0.994)	D	D (−3.65)
chr12:52163700	*SCN8A*	18	p.Pro1141Thr	c.3421C>A	D (0.046)	D(0.957)	D	D (−2.68)

Mutation Taster and Polyphen-2 are functional prediction scores in which increasing values indicate a more damaging effect, while for SIFT and PROVEAN decreasing values are damaging. Abbreviations: D, damaging or deleterious; ref, reference allele. Transcripts are Sodium channel α2-subunit; *SCN2A*: NM_001040143.1; Potassium channel, Kv3.2; *KCNC2*: NM_139137.3; and Sodium voltage-gated channel alpha subunit 8; *SCN8A*: NM_014191.3.
